# A Rare Case of Remittent Male Invasive Ductal Carcinoma With New Metastasis After Incomplete Adjuvant Therapy

**DOI:** 10.7759/cureus.50400

**Published:** 2023-12-12

**Authors:** Sanjana R Mada, Hein H Zay, Jared J Bies, Eyoab Massebo, Claudia Didia

**Affiliations:** 1 Internal Medicine, Texas Tech University Health Sciences Center El Paso, El Paso, USA

**Keywords:** medication nonadherence, metastasis, adjuvant therapy, invasive ductal carcinoma, male breast cancer

## Abstract

Breast cancer is a rare disease in men with many barriers to effective management such as limited research and treatment modalities. While the current standard of care utilizes mastectomy and axillary dissection with chemotherapy, clinicians must follow the female-staged breast cancer protocol, as there is no established regimen for men. In this case presentation, we report a 43-year-old male with a prior history of ER-positive invasive ductal carcinoma (IDC) who presented with a recurrent breast lesion. The patient had previously undergone left breast mastectomy with sentinel node biopsy with negative margins. The patient declined adjuvant chemotherapy and tamoxifen therapy after the initial dissection. Three years after the primary dissection, the patient presents with a breast lesion and metastasis to bilateral axillary lymph nodes, lungs, and spine. The diagnosis was supported by a right axillary biopsy which revealed an ER-positive and PR-positive lesion. We want to shed light on the importance of complete and thorough treatment of primary IDC in men while highlighting the implications of incomplete treatment. We hope that this clinical case will serve as a guide for physicians in promoting adjuvant treatments after primary tumor removal in male IDC.

## Introduction

Breast cancer in males is rare, accounting for less than 1% of all breast cancer cases [1-4]. Due to the low global prevalence of male breast cancer (MBC), a significant gap exists in the available literature addressing the details of this disease in men. The majority of research efforts are centered around female breast cancer treatments, thus limiting the scope of investigation for male patients. While insights gained from female breast cancer studies hold potential applicability to MBC, it is essential to acknowledge the constraints posed by sex-specific disparities, hormonal regulations, and distinct metabolic responses to treatments [2].

The incidence of MBC varies across racial and ethnic lines, with African-American men exhibiting higher rates than Caucasians, Hispanics, and Asian/Pacific Islanders [5]. Several risk factors are associated with MBC, including positive family history, conditions linked to abnormal estrogen-to-androgen ratios, radiation exposures, and germline mutations in BRCA2 [6,7]. Unfortunately, diagnosis of breast cancer in males often occurs at later stages than in females, contributing to the manifestation of advanced disease characteristics such as significant tumor size, lymph node involvement, and metastasis to diverse sites such as the lungs, bones, liver, and central nervous system [1,2,8,9]. The median age of diagnosis for men, approximately 65 years, is nearly a decade older than that for women [1]. Most cases of MBC are categorized as infiltrating ductal carcinoma, with a minority attributed to in situ carcinoma [1,2]. The typical hormonal profile includes the expression of the estrogen receptor (ER), progesterone receptor (PR), and androgen receptor (AR) [4,10]. Noteworthy clinical findings include nipple retraction, identification of a retroareolar mass during physical examination, and serosanguinous nipple discharge [1].

Early diagnosis and treatment significantly improve survival rates. However, due to the delayed diagnoses often observed in males, treatment approaches may be impacted. While mastectomy with axillary dissection remains the conventional treatment, locoregional radiotherapy might also be warranted [1]. Chemotherapy is recommended for men with axillary involvement or negative hormone receptor status. Alternatively, tamoxifen is a standard adjuvant treatment that may also be used to improve postoperative outcomes of the disease [1]. This study reports a case of a patient initially diagnosed with ER-positive invasive ductal carcinoma (IDC), subsequently undergoing mastectomy and sentinel node biopsy. Our patient experienced a recurrence of his initial cancer with extensive metastasis due to the decline of the chemotherapy regimen and incomplete outpatient follow-up.

## Case presentation

In December 2017, a 43-year-old male with a past medical history of supraventricular tachycardia, type II diabetes mellitus, hypertension, and dyslipidemia presented to the hospital for a left breast mass. The patient has a family history of breast, ovarian, colon, and pancreatic cancer; however, these instances were not observed among first-degree relatives. Upon admission, the recorded BMI was 39. His chief complaint included the presence of a lump within his left breast, which gradually increased in size over the course of six months, accompanied by nipple retraction. Subsequent physical examination showed a palpable, firm 2 cm mass located in the left retroareolar breast region, with the presence of left axillary lymphadenopathy.

**Figure 1:**
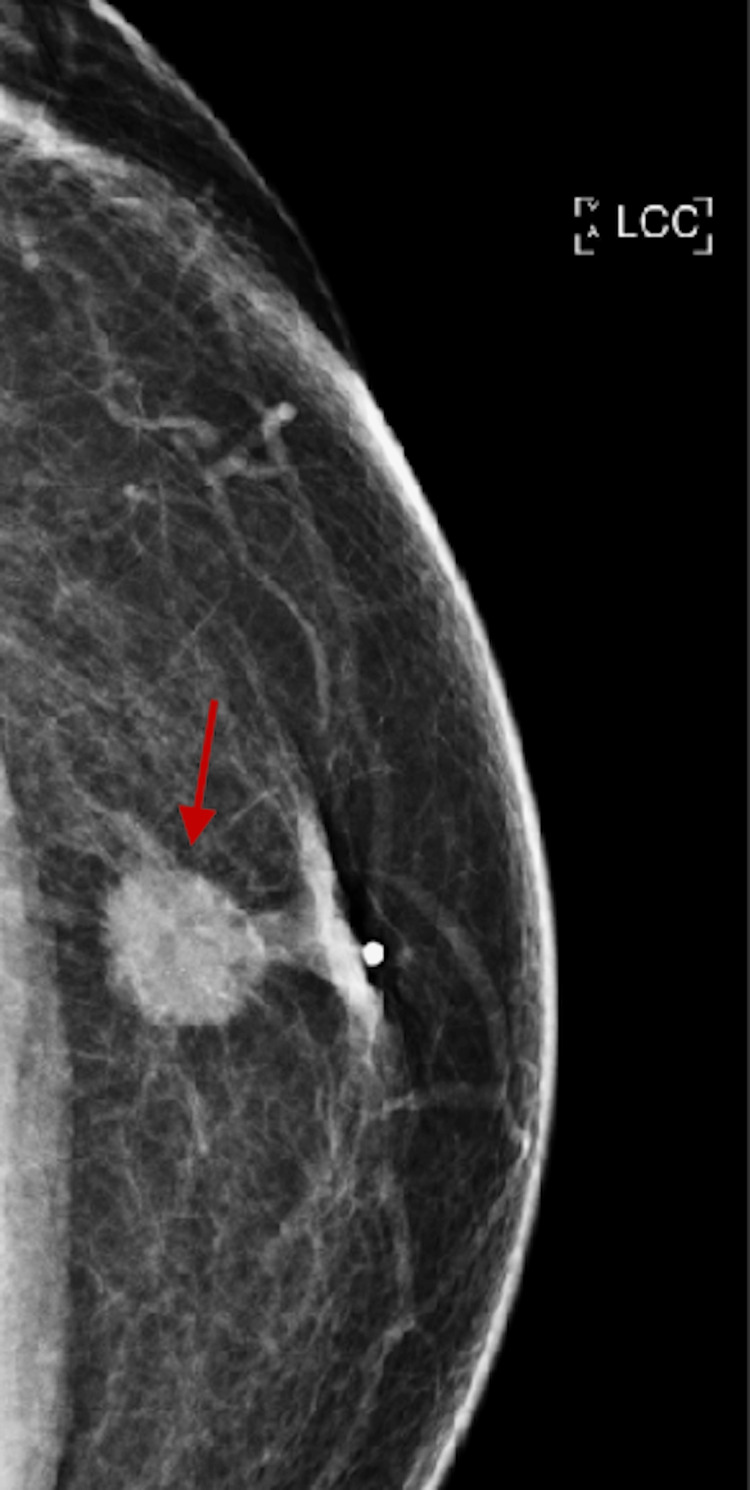
Diagnostic mammogram showing a 2 cm mass in the left retroareolar region

**Figure 2:**
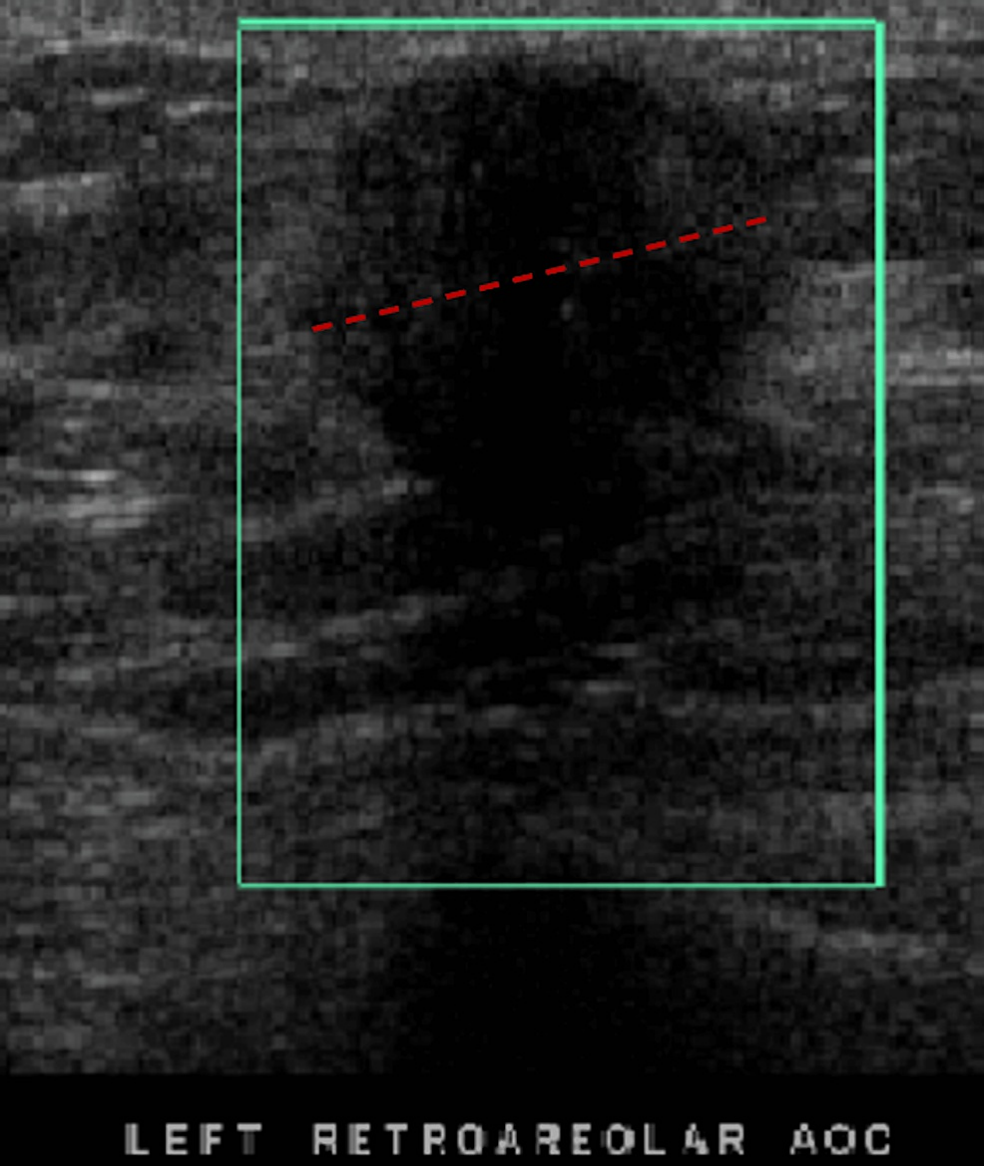
Ultrasound of the left retroareolar mass

Diagnostic mammography (Figure 1) demonstrated a 2 cm mass in the left retroareolar region with a few pleomorphic microcalcifications, nipple retraction, and mild left axillary lymphadenopathy. It was given a BI-RADS score of 5. These findings raised a strong suspicion of a metastatic lesion. An ultrasound of the left breast (Figure 2) also confirmed a 2.3 cm irregular mass concerning potential malignancy.

The final pathology report of the breast biopsy showed left breast IDC at least 2 cm in greatest dimension, Nottingham III, ER-positive 95%, PR-negative, and Her2-neu-negative (by FISH). Other imaging at the time included a full body bone scan and CT of the abdomen and pelvis which showed no other metastasis.

In February 2018, the patient underwent left simple mastectomy and left axilla sentinel biopsy. The conclusive pathology report showed a 2.3 cm IDC with resected margins free of invasive carcinoma. Notably, there was an absence of lymph vascular invasion, and a focal 0.4 cm ductal carcinoma in situ with high nuclear grade was present, though resected margins were negative for ductal carcinoma in situ. Examination of the sentinel lymph node excision revealed one lymph node negative for metastatic carcinoma, while a separate left axillary lipoma excision showed four lymph nodes negative for metastatic carcinoma. Following these procedures, the tumor's stage was designated as pT2N0(sn)MX. Subsequent laboratory analysis revealed an equivocal Her 2/neu status. Furthermore, genetic testing for BRCA1 and BRCA2 yielded negative results in our patient.

The patient's case was evaluated by an oncologist at a breast clinic to determine the appropriate treatment for his stage II IDC. Given the rarity of MBC and the absence of established treatment protocols, the NCCN guidelines advise adopting a similar approach as for stage II IDV in females. After receiving comprehensive education about the potential benefits, associated risks of chemotherapy, and hormonal treatment involving tamoxifen, the patient opted to decline further therapy. Additionally, it was recommended that the patient consult with a radiation oncologist to assess the suitability of radiotherapy and initiate tamoxifen treatment. However, the patient was subsequently lost to follow-up. 

**Figure 3:**
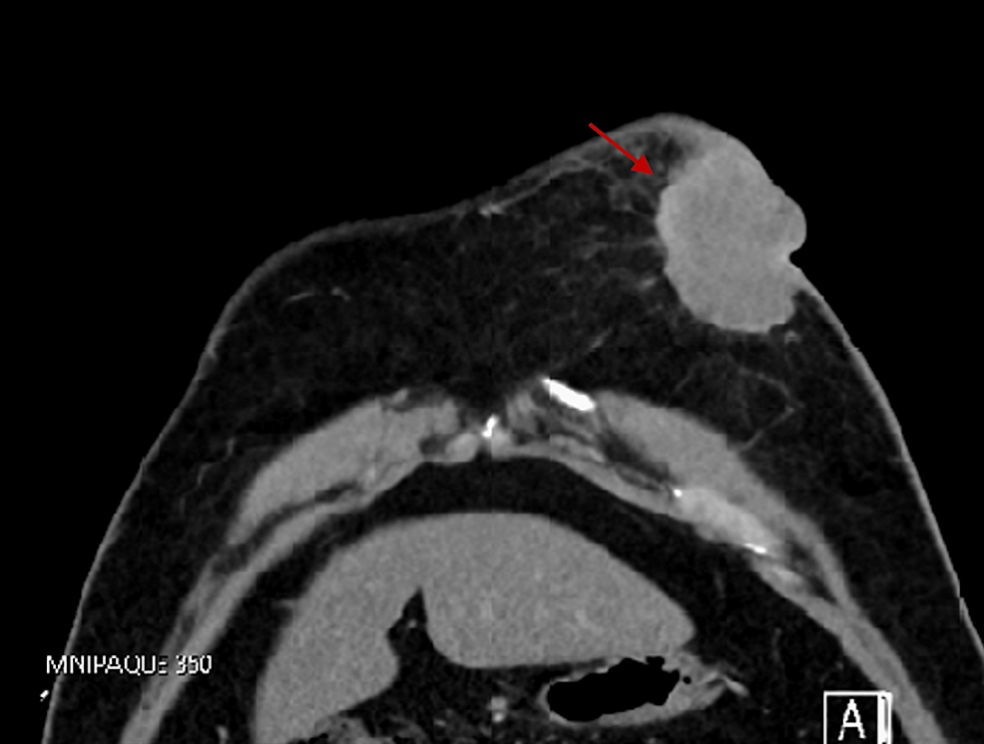
CT thorax showing a heterogeneous soft tissue mass

**Figure 4:**
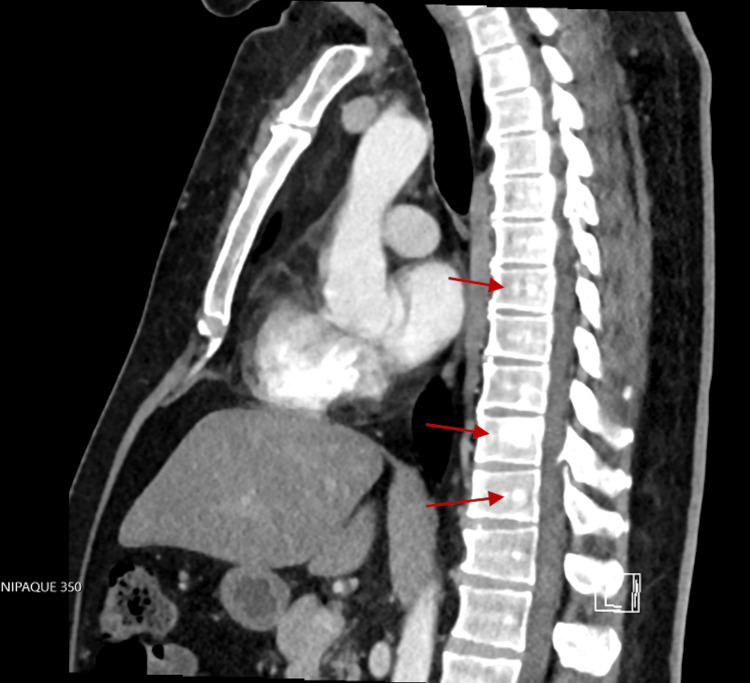
CT thorax showing sclerotic lesions in the spine

The patient returned to the hospital in August 2022 for heart palpitations and chest pain localized to the anterior chest with edema, erythema, and serosanguinous drainage from his previous mastectomy lesion. He also noticed a marble-sized mass in the right axilla. A CT thorax (Figure 3) was repeated and showed an irregularly shaped heterogeneous soft tissue mass in the left retroareolar region associated with bilateral axillary lymphadenopathy. It also showed multiple scattered sclerotic patchy areas are seen within the thoracic vertebrae due to potential metastasis (Figure 4). The CT abdomen was negative for metastasis. The patient was set up with an appointment for an ultrasound and biopsy at an outpatient breast clinic, but he did not attend. 

**Figure 5:**
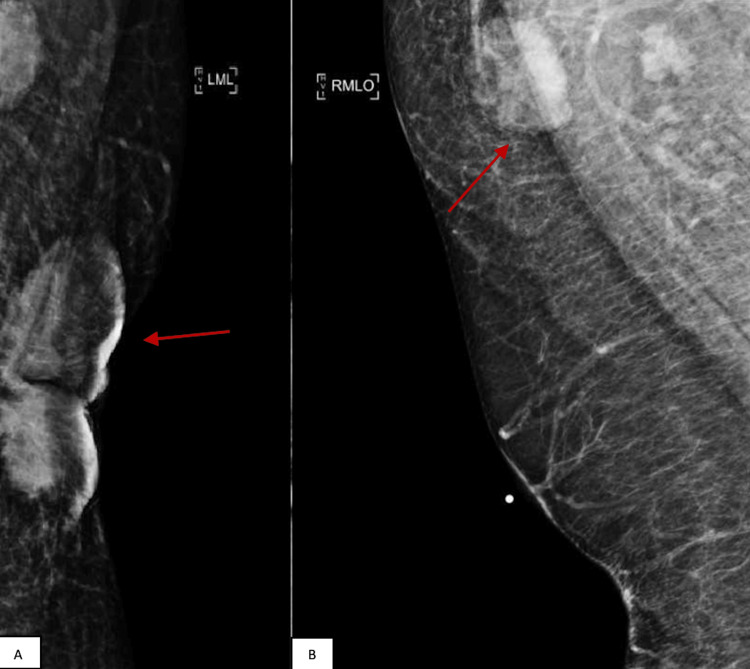
Diagnostic mammogram. (A) An irregular mass located in the left retroareolar breast. (B) Right axillary lymphadenopathy

**Figure 6:**
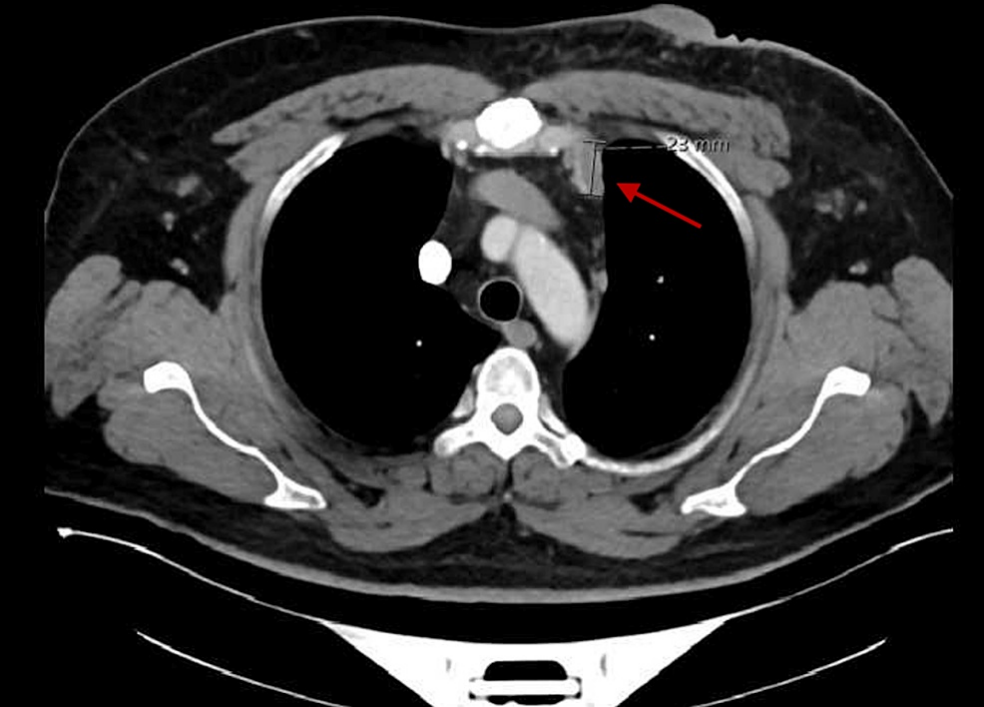
CT thorax showing an enlarged lymph node at the manubrium sternal junction

In February 2023, a diagnostic mammogram revealed a substantial irregular mass located in the retroareolar region of the left breast, accompanied by bilateral axillary adenopathy, and classified as a BI-RADS score of 5 (Figure 5). A thoracic CT scan indicated progression of the previously identified malignant mass within the left breast, along with advancing bilateral axillary lymphadenopathy. Furthermore, a newly enlarged lymph node emerged in the left internal mammary chain/left anterior mediastinum, situated at the level of the manubrium sternal junction (Figure 6). Multiple sclerotic lesions across the thoracic spine raised concerns about potential metastatic involvement (shown in Figure 4). Subsequent to this, another biopsy was conducted, this time in the right axillary region, which revealed positive ER and PR statuses, while HER2 status was negative.

The patient subsequently sought care with a breast oncologist after discharge from the hospital. Since there were no indications of visceral crisis during the visit, the patient's treatment regimen was initiated, consisting of tamoxifen 20 mg once a day and abemaciclib 150 mg twice a day. Additionally, due to bone metastasis, zoledronic acid (Zometa) infusion was administered.

## Discussion

MBC is an uncommon occurrence, accounting for merely 1% of all cancer diagnoses in males and contributing to less than 0.2% of cancer-related mortality attributed to breast cancer [1-4]. Many cases are typically diagnosed in stage III or IV due to delays in seeking medical attention for symptoms [3]. Only a handful of instances of MBC have been documented within the literature. While cases of MBC with metastatic progression remain relatively rare, they do exist [8,9]. Among these instances, the occurrences of male IDC presenting with metastasis have been reported. This limited published literature of MBC gives rise to a substantial gap in targeted research initiatives, particularly pertaining to preventive strategies and therapeutic interventions tailored to this unique subset of patients.

Certain ethnic groups such as African Americans have higher rates of MBC compared to Caucasians, Hispanics, or Asian/Pacific Islanders [5]. Various factors predispose males to MBC, while others exhibit protective effects. Risk factors include a first-degree family history of breast cancer, heightened estrogen exposure, hepatic dysfunction, obesity, orchitis, cryptorchidism, and BRCA gene mutations [11]. Changes within the adipose tissue microenvironment also merit consideration as MBC risk contributors, primarily due to hormonal therapy exposure [12]. No established correlation has been established between gynecomastia and heightened MBC risk [13]. In terms of protective factors, lifestyle differences such as an increased physical workload, displayed a decreased correlation with MBC risk [14]. It is imperative to highlight that our patient lacked any established risk factors for MBC development, with the exception of an elevated BMI of 39.

Since there is less mammary parenchyma in males compared to females, a comprehensive approach including clinical examination, mammography, cytology, and percutaneous biopsies is important [15]. A core needle biopsy gives further information on the hormonal profile of the disease and the expression of the ER, PR, and AR [4,10]. In this specific case, our patient had a thorough diagnostic process. It began with a physical examination upon admission, followed by a diagnostic mammogram, an ultrasound-guided biopsy, and a CT scan of the thorax and abdomen. Furthermore, additional pathology studies were conducted on the resected mass. The biopsy of the breast revealed left breast IDC, categorized as Nottingham Grade III. The tumor exhibited ER positivity at 95% while being negative for PR and Her2-neu (determined by FISH).

After surgical resection of the primary mass, adjuvant therapy with tamoxifen remains pivotal for male patients affected by endocrine-responsive disease [16]. Another option for men with a contraindication to tamoxifen may be a combination of an aromatase inhibitor and a GnRH agonist/antagonist [17]. However, the impact of adjuvant chemotherapy on overall survival in MBC cases is still not studied extensively. Despite these promising insights, the differences in disease response to varying treatment modalities need further investigation.

Following our patient's left simple mastectomy and left axillary sentinel biopsy, he was subsequently referred to a breast oncologist for ongoing treatment. Notably, our patient made the decision to opt out of adjuvant radiotherapy, chemotherapy, or tamoxifen-based treatment approaches. Despite the presence of resected margins and lymph node pathologies that tested negative for ductal carcinoma in situ, our patient returned four years later. This time, he presented with a recurrence of the left breast mass, which had progressed to metastasize within the lymph nodes, mediastinum, and spine.

Lack of medication adherence is linked to unfavorable health consequences and increased healthcare spending. The current scope of reported non-adherence rates in cancer patients is between 16 to 100% [18]. This case emphasizes the need to prioritize education and medical interventions to reduce the recurrence of MBC. Additionally, the assessment of the Oncotype DX Breast Recurrence Score (RS) in male patients takes on unique significance due to distinct interpretations and implications [19]. Further investigation is needed, given that the RS appears to correlate with mortality at a lower threshold in males compared to females. This suggests that male breast cancer may possess distinct biological characteristics and prognostic factors when contrasted with their female counterparts [19]. While clinical efforts revolve around treatment strategies and survival outcomes for breast cancer patients, it is important to explore the patient's individual considerations and apprehensions regarding treatment continuation. This multifaceted perspective on a comprehensive approach not only addresses medical treatment but also considers broader aspects of patient well-being.

## Conclusions

MBC faces limited treatment options due to current guidelines tailored around female protocols. The standard for treatment involves simple mastectomy and sentinel lymph node biopsy, with adjuvant therapies contingent on tumor staging. Our objective is to emphasize the significance of comprehensive follow-up, coupled with detailed medical education regarding the possible options available for the thorough treatment of invasive ductal carcinoma. By addressing these aspects, the potential for noncompliance lessens, subsequently reducing the risk of disease recurrence and progression.
